# MMP-9 Deletion Attenuates Arteriovenous Fistula Neointima through Reduced Perioperative Vascular Inflammation

**DOI:** 10.3390/ijms22115448

**Published:** 2021-05-21

**Authors:** Yu-Chung Shih, Po-Yuan Chen, Tai-Ming Ko, Po-Hsun Huang, Hsu Ma, Der-Cherng Tarng

**Affiliations:** 1 Division of Plastic and Reconstructive Surgery, Department of Surgery, Taipei Veterans General Hospital, Taipei 11217, Taiwan; rogerstonekimo@gmail.com (Y.-C.S.); sma@vghtpe.gov.tw (H.M.); 2 Institute of Clinical Medicine, National Yang Ming Chiao Tung University, Taipei 11221, Taiwan; huangbsvgh@gmail.com; 3 Institute of Clinical Medicine, National Yang Ming University, Taipei 11221, Taiwan; 4 Department of Surgery, School of Medicine, National Yang Ming Chiao Tung University, Taipei 112, Taiwan; 5 Bioinformatics Program, Taiwan International Graduate Program, Academia Sinica, Taipei 115, Taiwan; shepherd71c@gmail.com (P.-Y.C.); taiming23@gmail.com (T.-M.K.); 6 Institute of Bioinformatics and Systems Biology, National Yang Ming Chiao Tung University, Hsinchu 300, Taiwan; 7 Institute of Information Science, Academia Sinica, Taipei 115, Taiwan; 8 Department of Biological Science and Technology, National Yang Ming Chiao Tung University, Hsinchu 300, Taiwan; 9 Institute of Biomedical Sciences, Academia Sinica, Taipei 115, Taiwan; 10 Center of Intelligent Drug System and Smart Bio-devices (IDS2B), National Yang Ming Chiao Tung University, Hsinchu 300, Taiwan; 11 Department of Critical Care Medicine, Taipei Veterans General Hospital, Taipei 11217, Taiwan; 12 Department of Surgery, School of Medicine, National Defense Medical Center, Taipei 11490, Taiwan; 13 Division of Nephrology, Department of Medicine, Taipei Veterans General Hospital, Taipei 11217, Taiwan; 14 Institute of Physiology, National Yang Ming Chiao Tung University, Taipei 11221, Taiwan

**Keywords:** arteriovenous fistula, neointima, matrix metalloproteinase 9, vascular inflammation

## Abstract

Matrix metalloproteinase 9 (MMP-9) expression is upregulated in vascular inflammation and participates in vascular remodeling, including aneurysm dilatation and arterial neointima development. Neointima at the arteriovenous (AV) fistula anastomosis site primarily causes AV fistula stenosis and failure; however, the effects of MMP-9 on perioperative AV fistula remodeling remain unknown. Therefore, we created AV fistulas (end-to-side anastomosis) in wild-type (WT) and MMP-9 knockout mice with chronic kidney disease to further clarify this. Neointima progressively developed in the AV fistula venous segment of WT mice during the four-week postoperative course, and MMP-9 knockout increased the lumen area and attenuated neointima size by reducing smooth muscle cell and collagen components. Early perioperative AV fistula mRNA sequencing data revealed that inflammation-related gene sets were negatively enriched in AV fistula of MMP-9 knockout mice compared to that in WT mice. qPCR results also showed that inflammatory genes, including tumor necrosis factor-α (TNF-α), monocyte chemoattractant protein-1 (MCP-1), interleukin-6 (IL-6), intercellular adhesion molecule-1 (ICAM-1), and vascular cell adhesion molecule-1 (VCAM-1), were downregulated. In addition, Western blot results showed that MMP-9 knockout reduced CD44 and RAC-alpha serine/threonine-protein kinase (Akt) and extracellular signal-regulated kinases (ERK) phosphorylation. In vitro, MMP-9 addition enhanced IL-6 and MCP-1 expression in vascular smooth muscle cells, as well as cell migration, which was reversed by an MMP-9 inhibitor. In conclusion, MMP-9 knockout attenuated AV fistula stenosis by reducing perioperative vascular inflammation.

## 1. Introduction

Arteriovenous (AV) fistulas have been widely used in the past 50 years for better vascular access for hemodialysis than arteriovenous graft and cuffed catheters due to lower infection rates and complications [1,2]. However, AV fistulas are not without problems and only about 60% of AV fistulas are functional 12 months after creation [3,4,5]. Inadequate outward remodeling (OR) and neointimal hyperplasia lead to AV fistula failure [6]. Several vascular biological pathways that contribute to AV fistula neointima formation have been identified, including inflammation, uremia, hypoxia, shear stress, and thrombosis [2,7,8,9,10,11,12]. Nevertheless, there are still no effective pharmacological agents for improving AV fistula patency.

Matrix metalloproteinases (MMPs) play an essential role in vascular remodeling by not only degrading the extracellular matrix (ECM) to facilitate cell migration, but also modifying cytokines and growth factors [13,14,15,16,17]. MMP-9, initially termed 92-kDa type IV collagenase or gelatinase B, also degrades ECM through a large spectrum of physiological and pathophysiological processes involving tissue remodeling [18]. Matrix fragments released by MMP-9 influence angiogenesis and tumor growth and liberate vascular endothelial growth factor (VEGF) and transforming growth factor-β (TGF-β) [19,20]. MMP-9 expression is increased in rodent and human AV fistula models [21,22,23,24,25,26], and genetic MMP-9 polymorphism is associated with altered patency of hemodialysis AVFs [27]. The tumor necrosis factor-α (TNF-α)–MMP-9–vascular smooth muscle cell (VSMC) migration axis contributes to neointima growth in a murine arterial injury model [28]. Simvastatin, which decreases both MMP-9 and VEGF-A expression, decreased neointima size and increased the lumen area in murine AV fistulas [29]. However, doxycycline, a general MMP inhibitor, did not significantly affect neointima size or AV fistula patency despite the suppression of MMP-9 expression [30]. The effects of MMP-9 on vascular remodeling of AV fistula do not seem to be identical to those on arterial injury lesions. In our previous report, MMP-9 mRNA expression in AV fistula was upregulated in chronic kidney disease (CKD) condition and reduced after lowering indoxyl sulfate, a uremic toxin, with oral carbonaceous adsorbent [31]. The surge of MMP-9 was also associated with the increase of neointima size. Thus, we used MMP-9 knockout mice with CKD to examine the role of MMP-9 in vascular remodeling after AV fistula creation.

## 2. Results

### 2.1. Neointima Increases Progressively in the AV Fistula Venous Segment of Wild-Type (WT) Mice

**Figure 1:**
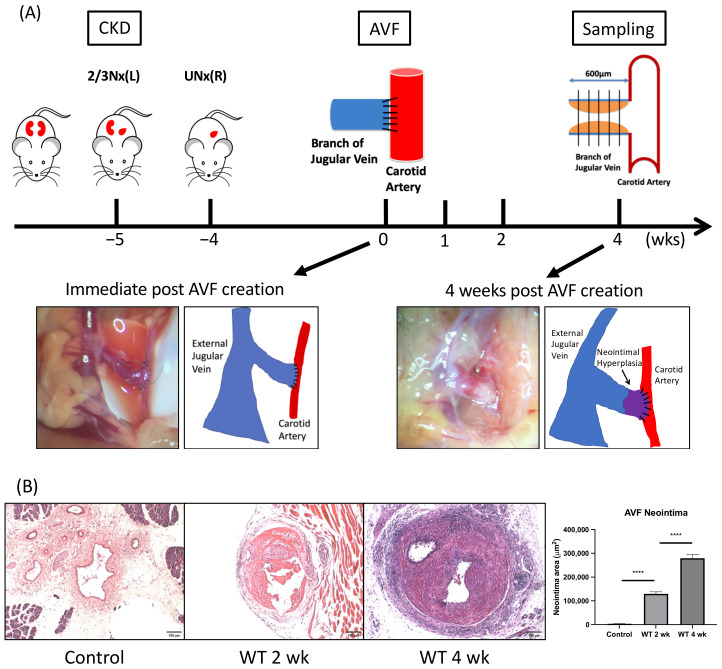
Neointima formation in the venous outflow tract of arteriovenous fistula (AVF). (**A**) Wild-type (WT) mice underwent 5/6 nephrectomy for chronic kidney disease (CKD) induction, followed by AVF creation four weeks later. Grossly, neointima formation was observed at proximal AVF venous segment. (**B**) Histologically, the initial 600 μm venous outflow tract was divided into five 100 μm thick segments as illustrated in (**A**), scale bar = 100 μm. After elastin staining, neointima at each segment was examined, which was found to enlarge gradually after AVF creation. **** *p* < 0.0001. Data presented as mean ± SEM and analyzed using one-way analysis of variance (ANOVA) followed by Bonferroni post hoc analysis; *n* = 6–7 in each group. AVF, arteriovenous fistula; CKD, chronic kidney disease; 2/3 Nx, 2/3 nephrectomy; UNx, uninephrectomy; WT, wild-type.

Four weeks after completing subtotal nephrectomy, an AV fistula was created at the end of the dorsomedial branch of the external jugular vein (EJV) anastomosing to the side of the common carotid artery (CCA) at the right neck (Figure 1A, lower panel left). Four weeks after its creation, neointima was observed at the proximal venous segment of AV fistula (Figure 1A, lower panel right). Histologically, neointima developed in the AV fistula venous segment two weeks after AVF formation compared to the contralateral control vein (129,985 ± 8328 μm^2^ vs. 3694 ± 171.1 μm^2^, *p* < 0.001). Neointima size further increased four-weeks post AVF creation compared to 2 weeks (279,036 ± 15,650 μm^2^ vs. 129,985 ± 8328 μm^2^, average increase 115%, *p* < 0.001; *n* = 6–7 at each time point; Figure 1B).

### 2.2. Vascular Inflammation Was Detected in the Early Perioperative Phase of AV Fistula

**Figure 2:**
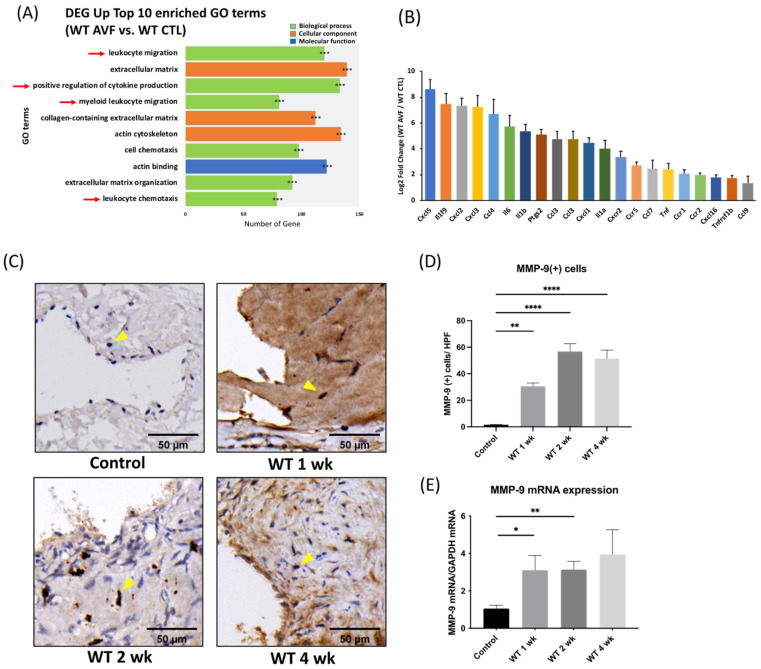
Vascular inflammation and increased matrix metalloproteinase 9 (MMP-9) expression in AVF venous segment. (**A**) RNA-seq data analysis revealed four out of the top 10 enriched gene ontology terms were related to inflammation. In wild-type (WT) AVF compared to contralateral veins sampled 1-week post AV fistula creation. *** *p adjust* < 0.001 (**B**) The expression of multiple proinflammatory genes, including chemokines and cytokines, was also elevated (*n* = 3 in each group). (**C**,**D**) Using immunohistochemical staining, MMP-9(+) cells in neointima lesions increased significantly after AV fistula surgery, scale bar = 50 μm. (*n* = 6–7 in each time point). (**E**) MMP-9 mRNA expression was upregulated at the early perioperative phase as well. (*n* = 6–7 in each time point). * *p* < 0.05, ** *p* < 0.01, **** *p* < 0.0001. Data presented as mean ± SEM and analyzed by one-way analysis of variance (ANOVA) followed by Bonferroni post hoc analysis. CTL, control; DEG, differentially expressed genes; GO, gene ontology.

Genome-wide gene expression data of the AV fistula venous segments and contralateral jugular veins were obtained after sequencing the mRNA (RNA-seq), which underwent differential gene expression analysis and functional enrichment. One week after AV fistula creation, significant vascular inflammation was observed by comparing AV fistula venous segments and contralateral control veins in the early perioperative phase. A total of four out of the top 10 significantly enriched gene ontology (GO) terms were related to inflammation, including leukocyte migration, positive regulation of cytokine production, myeloid leukocyte migration, and leukocyte chemotaxis, padj < 0.001 in all top 10 GO terms (Figure 2A); *n* = 3. Among the rest, three terms were related to ECM remodeling and cell migration each. Additionally, the expression of multiple proinflammatory genes, including chemokines and cytokines, was also elevated, with padj < 0.05 in all listed genes (Figure 2B).

### 2.3. MMP-9 Expression Elevates Significantly during the Perioperative Period after AV Fistula Creation

After AV fistula creation, MMP-9(+) cells increased significantly in the AV fistula venous segment. The number of MMP-9(+) cells within neointima lesions increased from 1.57 ± 0.20/high power field (HPF) in the unoperated control veins to 30.50 ± 2.55, 56.71 ± 5.94, 51.33 ± 6.51/HPF one, two, and four weeks after AVF creation, respectively, in the AV fistula venous segment (*p* = 0.0011, <0.0001, and <0.0001, respectively; Figure 2C,D). qPCR results indicated that MMP-9 mRNA expression in the AV fistula venous segment increased and reached significance in 1- and 2-weeks post AV fistula creation compared to that in the unoperated control veins (1 week 3.1 ± 0.79, 2 weeks 3.13 ± 0.45, 4 weeks 3.95 ± 1.31 vs. control vein 1.05 ± 0.17; *p* = 0.0458, 0.0031, 0.0958, respectively; Figure 2E).

### 2.4. MMP-9 Knockout Does Not Have Any Detrimental Influence on AVF Venous Segment OR, but Attenuates Neointimal Hyperplasia and Increases Lumen Area

**Figure 3:**
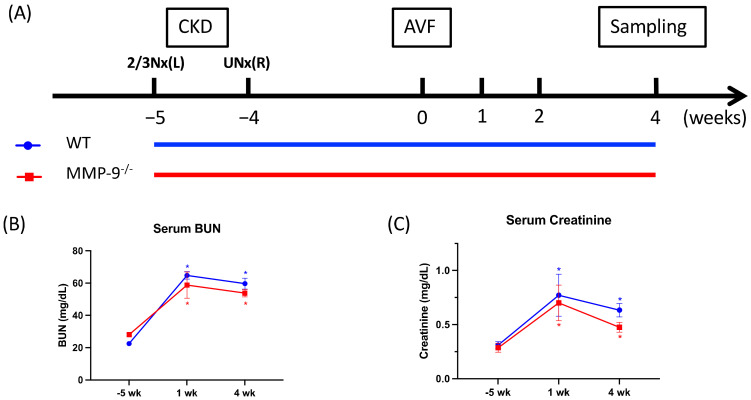

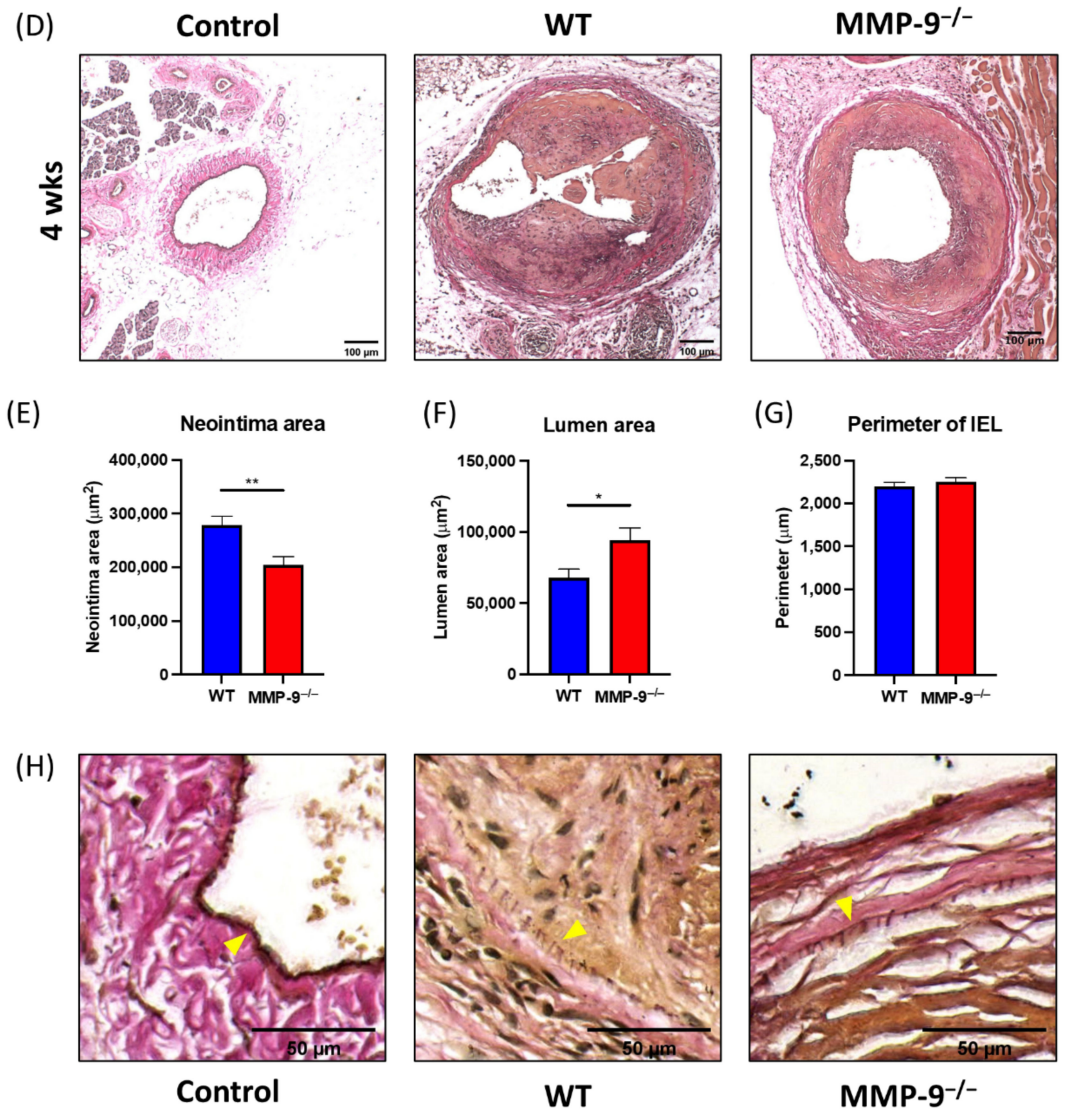
Matrix metalloproteinase 9 (MMP-9) knockout attenuated neointima formation and increased lumen size in arteriovenous (AV) fistula venous segment. (**A**) AV fistula was created in both wild-type (WT) and MMP-9^−/−^ mice to examine the influence on AV fistula. (**B**,**C**) Chronic kidney disease was successfully induced in both WT and MMP-9^−/−^ mice with significantly elevated serum blood urea nitrogen (BUN) and creatinine. (**D**) Morphometric analysis was done after elastin staining, scale bar = 100 μm. (**E**) Neointima size decreased and (**F**) lumen area enlarged after MMP-9 knockout (*p* = 0.0013 and 0.0116, respectively). (**G**) Internal elastic lamina (IEL) perimeter was not significantly different between WT and MMP-9^−/−^ mice. (**H**) IEL structure was examined under high magnification, and loose structure was found in both WT and MMP-9^−/−^ mice AV fistula venous segment, scale bar = 50 μm. No visible detrimental effect was detected after MMP-9 knockout. * *p* < 0.05, ** *p* < 0.01. Data presented as mean ± SEM. Data analyzed by Student’s *t*-test one-way, *n* = 6–7 in each group.

We further investigated the effect of MMP-9 on AVF stenosis in MMP-9 knockout mice [32]. AVF was created in both WT and MMP-9^−/−^ mice after CKD induction by subtotal nephrectomy (Figure 3A). The CKD conditions were successfully created in WT and MMP-9^−/−^ mice with significantly elevated serum blood urea nitrogen (BUN) and creatinine levels (Figure 3B,C).

The AVF venous segment was morphometrically analyzed by staining the sections with Weigert elastin (Figure 3D). The internal elastic lamina (IEL) was identified and traced to separate neointima from the remaining venous vascular structures. The neointima within the AV fistula venous segment caused stenosis, and IEL perimeter represented the outward expansion of vein under high-pressure flow from the artery [33]. AV fistula patency depends on the balance between venous wall OR and luminal narrowing due to neointimal hyperplasia [6]. Four weeks after AVF creation, neointima within the AVF venous segment was significantly reduced in MMP-9^−/−^ mice compared with that in WT mice (204,361 ± 15,511 μm^2^ vs. 279,036 ± 15,650 μm^2^, *p* = 0.0013; Figure 3E). The lumen area of the AVF venous segment in MMP-9^−/−^ mice was also significantly larger than that in WT mice 4-weeks post AVF creation (94,325 ± 8515 μm^2^ vs. 67,957 ± 5910 μm^2^, *p* = 0.0116; Figure 3F). However, the IEL perimeter in WT and MMP-9^−/−^ mice showed no difference 4-weeks post AVF creation (2200 ± 47.44 μm vs. 2253 ± 48.55 μm, *p* = 0.4398; Figure 3G). Thus, MMP-9 knockout did not hamper venous OR. Under high-power magnification, a loose IEL structure was found in both WT and MMP-9^−/−^ mice AV fistula venous segments compared to the more compact structure in the unoperated contralateral control vein (Figure 3H).

### 2.5. MMP-9 Knockout Significantly Diminished α-Smooth Muscle Actin (α-SMA) and Collagen Content in Neointima

**Figure 4:**
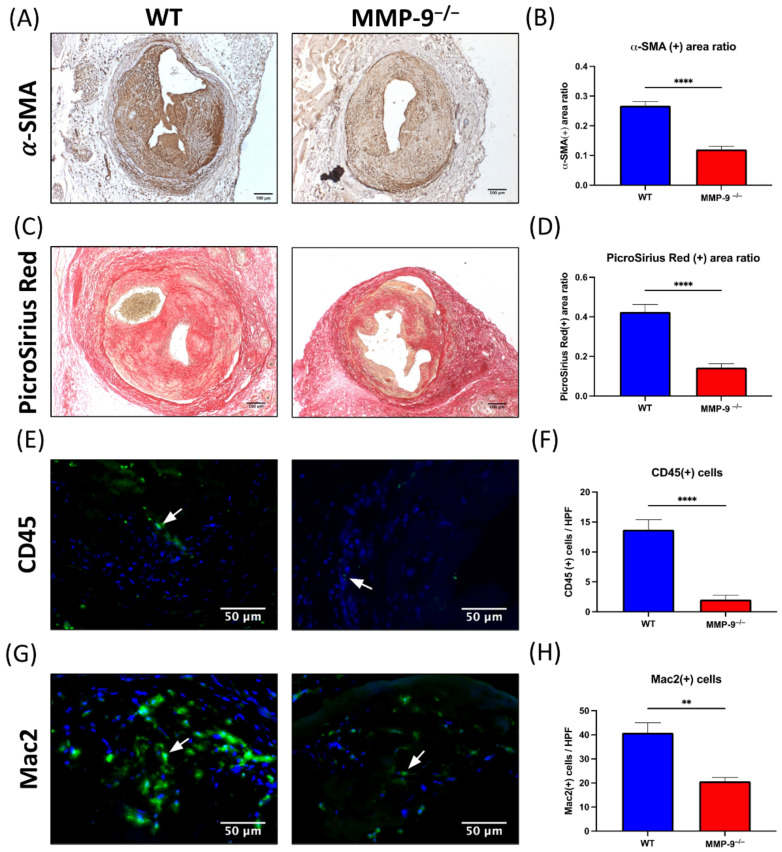
Matrix metalloproteinase 9 (MMP-9) knockout reduced α-smooth muscle actin (α-SMA) antibody and Picro Sirius stained area, and CD45(+) and Mac2(+) cells in neointima of AV fistula venous segment. (**A**) α-SMA(+) cells were the major cellular component within neointima. (**B**) The α-SMA(+) area ratio in neointima reduced after MMP-9 knockout (*p* < 0.0001). (**C**) Extracellular component of neointima was assessed with Picro Sirius Red staining. (**D**) The Picro Sirius Red(+) area ratio within neointima decreased in MMP-9^−/−^ mice (*p* < 0.0001). (**E**) Leukocyte infiltration within neointima was determined by CD45 immunofluorescence. (**F**) MMP-9 deletion decreased CD45(+) cells/HPF in neointima (*p* < 0.0001). (**G**) Macrophages in the neointima was examined by Mac2 immunofluorescence. (**H**) The number of Mac2(+) cells/HPF decreased in MMP-9^−/−^ mice (*p* = 0.0011). White arrows indicated positively stained cells. ** *p* < 0.01, **** *p* < 0.0001. Data presented as mean ± SEM. Data analyzed by Student’s *t*-test one-way, *n* = 6–7 in each group. HPF, high power field.

Immunohistochemical staining with α-SMA antibody and Picro Sirius red staining was used to identify the change in specific compositions within neointima tissue. The neointimal major cellular component was α-SMA(+) cells, mainly comprising vascular smooth muscle cells and myofibroblasts. The α-SMA(+) area ratio in MMP-9^−/−^ mice neointima was significantly smaller than that in WT mice 4 weeks after AV fistula creation (0.121 ± 0.010 vs. 0.268 ± 0.014, *p* < 0.0001, Figure 4A,B).

The extracellular component within the neointima was assessed by collagen staining using Picro Sirius red. Despite the lack of MMP-9 expression, the collagen stained area ratio within the neointima in MMP-9^−/−^ mice was significantly reduced 4-weeks post AV fistula creation in comparison with that in WT mice (0.144 ± 0.019 vs. 0.425 ± 0.037, *p* < 0.0001, Figure 4C,D).

### 2.6. MMP-9 Knockout Reduced CD45(+) and Mac2(+) Cells within Neointima

Immunofluorescence staining with CD45 and Mac2 antibodies was used to determine leukocyte and macrophage infiltration within the neointima [34]. Two weeks after AV fistula creation, CD45(+) cells within neointima decreased in MMP-9^−/−^ mice compared to that in WT mice (2.04 ± 0.73 cells vs. 13.7 ± 1.69 cells/HPF, *p* < 0.0001, Figure 4E,F). Mac2(+) cells represented macrophage infiltration within neointima, which was reduced by MMP-9 deficiency 2-weeks post AV fistula creation (20.67 ± 1.65 cells vs. 40.86 ± 4.16 cells/HPF, *p*= 0.0011, Figure 4G,H). Therefore, MMP-9 knockout decreased inflammatory cell infiltration within the neointima of AV fistula venous segment during the perioperative phase.

### 2.7. MMP-9 Knockout Downregulates Perioperative Inflammation in AV Fistula

**Figure 5:**
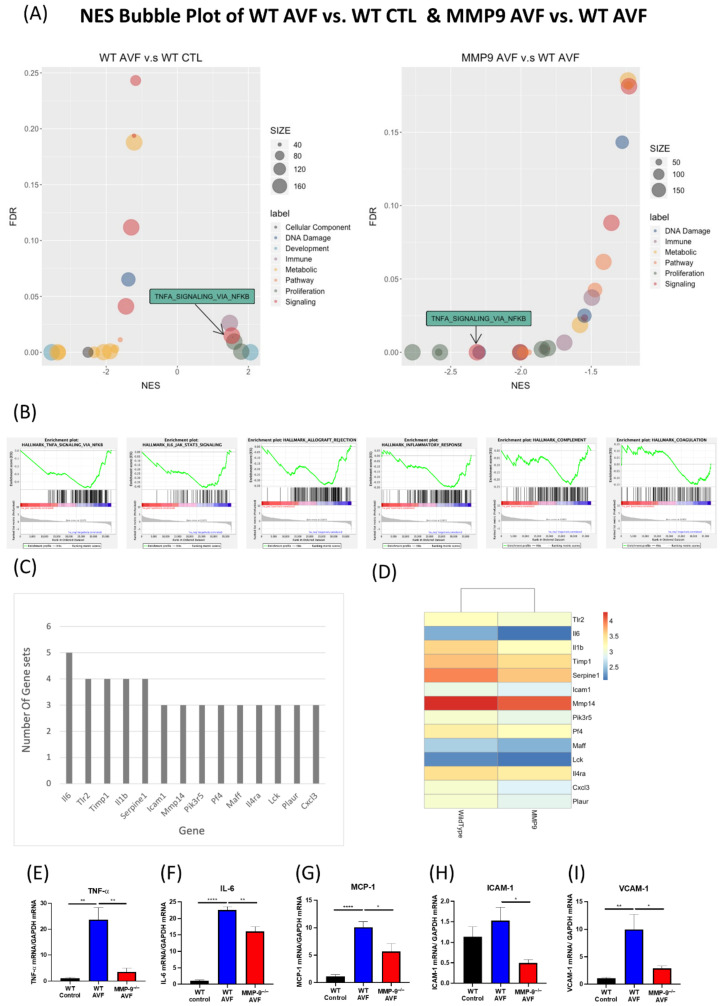
Genome-wide mRNA expression difference of WT and MMP-9^−/−^ mice AV fistulas. (**A**) Gene set enrichment analysis (GSEA) with Hallmark gene sets was performed to analyze the differentially expressed genes between WT and MMP-9^−/−^ mice. Significantly enriched pathways in different categories are visualized using bubble plots of normalized enrichment score (NES) for different comparisons, including WT AVF vs. WT CTL and MMP9 AVF vs. WT AVF, and the size of each bubble is proportional to the number of core genes within the pathway. Only pathways with FDR < 0.25 were included. TNFA_SIGNALING_VIA_NFKB was positively enriched in WT AVF vs. WT CTL, but negatively enriched in MMP9 AVF vs. WT AVF. (**B**) GSEA enrichment plots of immune-related pathways in MMP-9^−/−^ AVF vs. WT AVF. (**C**) Leading-edge analysis of six immune-related pathways. (**D**) The normalized expression value of these 14 key driver genes were expressed in heatmap, *n* = 3 in each group. Expression of inflammation-related genes usually associated with AV fistula stenosis such as (**E**) TNF-α, (**F**) IL-6, (**G**) MCP-1, (**H**) ICAM-1, and (**I**) VCAM-1, were examined by qPCR, *n* = 6–7 in each group. * *p* < 0.05, ** *p* < 0.01, **** *p* < 0.0001. Data presented as mean ± SEM. Data analyzed by one-way analysis of variance (ANOVA) followed by Bonferroni post hoc analysis. ICAM-1, intercellular adhesion molecule 1; IL-6, interleukin 6; MCP-1, monocyte chemoattractant protein 1; NES, normalized enrichment score; NFKB, nuclear factor kappa-light-chain-enhancer of activated B cells; TNFA and TNF-α, tumor necrosis factor-α; VCAM-1, vascular cell adhesion molecule 1.

We further evaluated the genome-wide mRNA expression differences between AV fistula and contralateral control vein of WT mice as well as between AV fistulas of WT and MMP-9^−/−^ mice using gene set enrichment analysis (GSEA) [35,36]. The Hallmark gene set collection from the molecular signature database was used to provide transcription profiling [35,37] and significantly enriched pathways of different gene set categories. The normalized enrichment score (NES) and false discovery rate (FDR) of each gene sets were visualized using bubble plots of different comparisons in Figure 5A. The GSEA results are provided in Table S1. Among these, the enrichment pattern of TNFA_SIGNALING_VIA_NFKB correlated the change of venous stenosis in AVF. Since this pathway is a major inflammatory pathway, we further examined five other immune-related pathways in the Hallmark gene set collection with similar enrichment patterns, including ALLOGRAFT_REJECTION, COMPLEMENT, INFLAMMATORY_RESPONSE, IL6_JAK_STAT3_SIGNALING, and COAGULATION (Figure 5B). Then, leading-edge analysis was performed in these six immune-related pathways to explore the possible key driver genes among them. Only genes that recurred in more than three gene sets were selected, and IL-6 showed the highest number of recurrence (Figure 5C). We then utilized the normalized expression values of these 14 leading-edge genes to plot an expression heatmap, which showed that their expression increased in WT AV fistula samples (Figure 5D). Furthermore, we examined proinflammatory gene expression using qPCR, including TNF-α, interleukin-6 (IL-6), monocyte chemoattractant protein-1 (MCP-1), intercellular adhesion molecule-1 (ICAM-1), and vascular cell adhesion molecule-1 (VCAM-1), which are linked to neointima formation in AV fistula (Figure 5E–I). Since MMP-9 expression significantly increased in the early perioperative phase after AVF creation, we examined gene expression 1-week post-surgery. MMP-9 knockdown reversed the increased TNF-α expression in AVF venous segment 1-week post AVF creation (WT control 1.09 ± 0.24 vs. WT AVF 23.68 ± 4.59 vs. MMP-9^−/−^ AVF 3.54 ± 1.37; *p* = 0.001 and 0.0011, respectively; Figure 5C). IL-6 and MCP-1 expression pattern was similar to that of TNF-α (IL-6: 1.10 ± 0.21 vs. 22.56 ± 0.97 vs. 16.01 ± 1.51, *p* < 0.0001 and *p* = 0.0014; MCP-1: 1.16 ± 0.29 vs. 10.10 ± 1.05 vs. 5.70 ± 1.40, *p* < 0.0001 and *p* = 0.0209; Figure 5D,F). MMP-9 knockdown also downregulated the mRNA expression of ICAM-1 and VCAM-1, two cell adhesion molecules usually upregulated in vascular inflammation (ICAM-1: 1.14 ± 0.24 vs. 1.53 ± 0.7 vs. 0.50 ± 0.09, *p* = 0.224 and 0.0224; VCAM-1: 1.07 ± 0.17 vs.9.93 ± 2.78 vs. 2.91 ± 0.42, *p* = 0.0046 and 0.0238; Figure 5G,H).

### 2.8. MMP-2 Expression in AV Fistula Venous Segment Was Not Significantly Altered in MMP-9 Knockout Mice

**Figure 6:**
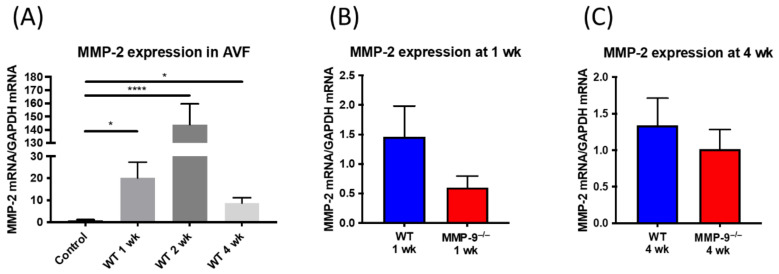
MMP-2 mRNA expression in AV fistula venous segment. (**A**) MMP-2 mRNA expression elevated after AV fistula creation comparing to contralateral veins sampled at 1 week, 2 weeks, and 4 weeks after AV fistula creation and the peak was at 2 weeks (*n* = 6~7 in each time point). (**B,C**) The deletion of MMP-9 did not make compensatory elevation of MMP-2 at 1-week and 4-weeks post AVF creation. (*n* = 6–7 in each group). * *p* < 0.05, **** *p* < 0.0001. Data presented as mean ± SEM and analyzed by one-way ANOVA followed by Bonferroni post hoc analysis for multiple comparison and Student’s *t*-test for comparison of two.

After AV fistula creation, MMP-2 expression surged at 1 week, 2 weeks and went down at 4 weeks after the surgery (1 week 20.11 ± 7.15, 2 weeks 143.8 ± 0.45, 4 weeks 8.7 ± 2.44 vs. control vein 0.98 ± 0.26; *p* = 0.0233, <0.0001, 0.0103, respectively), Figure 6A. Though both belonging to gelatinases, the deletion of MMP-9 did not make compensatory elevation of MMP-2 at 1-week and 4-weeks post AVF creation (WT vs. MMP-9^−/−^ 1 week 1.46 ± 0.52 vs. 0.61 ± 0.19, *p* = 0.1523; 4 weeks 1.34 ± 0.37 vs. 1.02 ± 0.27, *p* = 0.4835), Figure 6B,C.

### 2.9. MMP-9 Knockout Downregulated CD44 and RAC-Alpha Serine/Threonine-Protein Kinase (Akt) and Extracellular Signal-Regulated Kinase (ERK) Phosphorylation in AV Fistula Venous Segment

**Figure 7:**
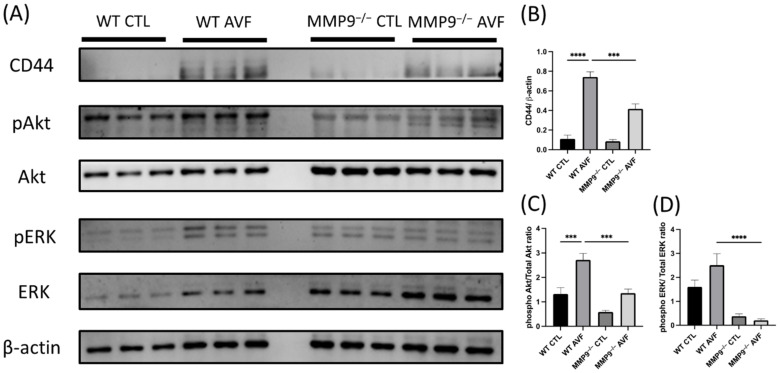
Matrix metalloproteinase 9 (MMP-9) knockout downregulated CD44 and RAC-alpha serine/threonine-protein kinase (Akt) and extracellular signal-regulated kinases (ERK) phosphorylation in arteriovenous (AV) fistula venous segment. (**A**) Western blotting was done for evaluating the protein expression in AV fistula venous segment. (**B**) One-week post AV fistula creation, CD44 expression was increased in wild-type (WT) mice, which was reversed by MMP-9 knockout. (**C**) Akt phosphorylation was also increased after AV fistula creation and attenuated by MMP-9 knockout. (**D**) Phospho-ERK level was significantly decreased in MMP-9^−/−^ mice. *** *p* < 0.001, **** *p* < 0.0001. Data presented as mean ± SEM. Data analyzed by one-way analysis of variance (ANOVA) followed by Bonferroni post hoc analysis, *n* = 6 in each group. CTL, control.

Protein expression in the AV fistula venous segment one week after its creation was confirmed by Western blotting (Figure 7A). CD44 is a major trigger of inflammation in AV fistulas [38]. In the current AV fistula model, CD44 was elevated after AV fistula creation in WT mice, and the surge was attenuated in MMP-9^−/−^ mice (WT CTL 0.16 ± 0.06 vs. WT AVF 0.81 ± 0.11, MMP-9^−/−^ CTL 0.18 ± 0.09 vs. 0.43 ± 0.04; *p* < 0.0001 and *p* = 0.0123, respectively; *n* = 6 in each group; Figure 7B). In addition to CD44, the Akt and ERK phosphorylation is also linked to AV fistula stenosis [12,39]. Phospho-Akt was significantly upregulated after AV fistula surgery and MMP-9 deficiency ameliorated the effect (WT CTL 1.32 ± 0.26 vs. WT AVF 2.72 ± 0.26, MMP-9^−/−^ CTL 0.59 ± 0.06 vs. MMP-9^−/−^ 1.36 ± 0.17; *p* = 0.0006 and 0.0009 respectively; Figure 7C). Moreover, ERK phosphorylation level was significantly lower in MMP-9^−/−^ mice (WT CTL 1.61 ± 0.28 vs. WT AVF 2.51 ± 0.48, MMP-9^−/−^ CTL 0.38 ± 0.10 vs. MMP-9^−/−^ AVF 0.21 ± 0.05; *p* < 0.0001 for WT AVF vs. MMP-9^−/−^ AVF; Figure 7D).

### 2.10. MMP-9 Increases MCP-1 and IL-6 Expression and Migration in VSMCs

**Figure 8:**
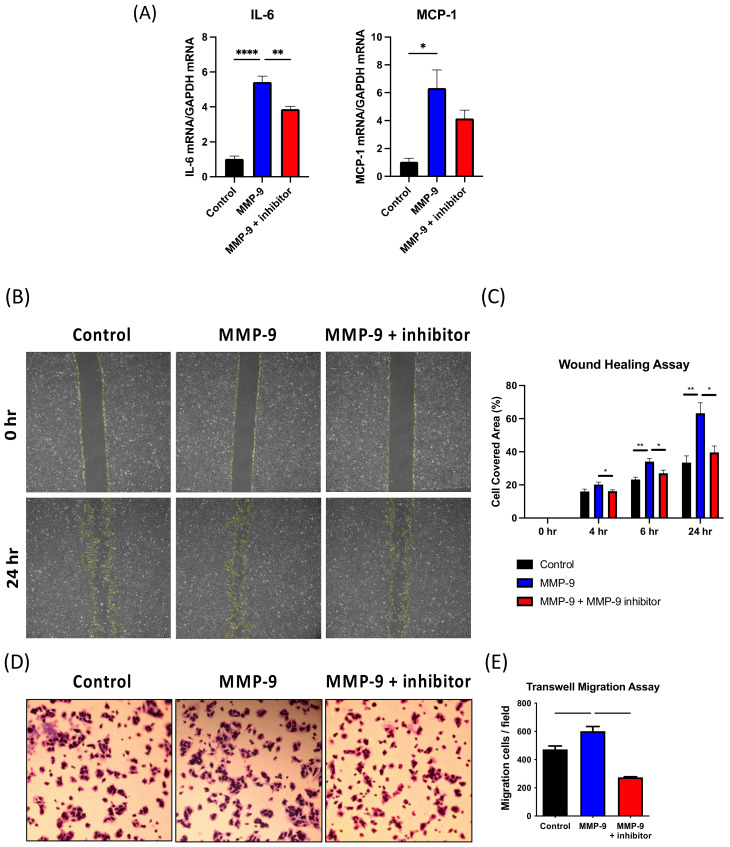
Mouse vascular smooth muscle cell line (MOVAS) was used for in vitro study. (**A**) MCP-1 and IL-6 mRNA expression was assessed by qPCR. The influence of matrix metalloproteinase 9 (MMP-9) on MOVAS migration was assessed using (**B**,**C**) wound healing and (**D**,**E**) transwell migration assays. * *p* < 0.05, ** *p*< 0.01, *** *p* < 0.001, **** *p* < 0.0001. Data presented as mean ± SEM. Data analyzed by one-way analysis of variance (ANOVA) followed by Bonferroni post hoc analysis, *n* = 6 in each group.

VSMCs are a major cellular component in the AV fistula neointima as reported using lineage tracing. Therefore, the mouse vascular smooth muscle cell line (MOVAS) was used to examine the effects of MMP-9 on neointima formation in AV fistula in vitro. MOVAS was treated with mouse recombinant MMP-9 protein, which significantly upregulated MCP-1 and IL-6 mRNA expression. IL-6 expression in Control-, MMP-9-, and MMP-9 + inhibitor-treated cells was 1.03 ± 0.15, 5.43 ± 0.33, and 3.89 ± 0.15, respectively (*p* < 0.001 and *p* = 0.009, respectively), while MCP-1 expression in Control-, MMP-9-, and MMP-9 + inhibitor-treated cells was 1.05 ± 0.25, 6.34 ± 1.30, and 4.15 ± 1.06, respectively (*p* = 0.013 for Control vs. MMP-9) (Figure 8A). Wound healing assay showed that MMP-9 enhanced MOVAS migration and cell covered area, which were ameliorated by MMP-9 inhibitor (Control vs. MMP-9 vs. MMP-9 + inhibitor at 4 h: 16.0 ± 1.45 vs. 20.20 ± 1.50 vs. 16.20 ± 0.86, *p* = 0.0491 for MMP-9 vs. MMP-9 + inhibitor, 6 h: 23.20 ± 1.50 vs. 34.00 ± 1.95 vs. 27.00 ± 1.93, *p* = 0.0023 and 0.0354 respectively, 24 h 33.40 ± 4.07 vs. 63.20 ± 6.40 vs. 39.60 ± 3.93, *p* = 0.0022 and 0.0108 respectively; *n* = 5 in each group; Figure 8B,C). The transwell migration assay also revealed similar results. MMP-9 administration increased the number of transwell migration cells, and the MMP-9 inhibitor reversed the condition (Control vs. MMP-9 vs. MMP-9 + inhibitor, 472.3 ± 24.25 vs. 601 ± 33.61 vs. 274 ± 4.58; *p* = 0.0276 and 0.0002, respectively; *n* = 3 in each group; Figure 8D,E). Taken together, MMP-9 elevated IL-6 and MCP-1 expression in vascular smooth muscle cells, which concurred with the proinflammatory role in the AV fistula model. The increased MOVAS migration by MMP-9 also partly explained its association with aggravated neointimal growth.

## 3. Discussion

The increased MMP-9 expression after AV fistula surgery can be due to several factors, including shear stress, uremic environment, inflammation, and hypoxia. Unbalanced matrix deposition and degradation affect vascular wall remodeling and are potentially related to neointima formation [40]. In this study, we used MMP-9 knockout mice to identify the impact of MMP-9 on AV fistula and confirmed that it reduced perioperative vascular inflammation and neointima formation in AV fistula venous segment. Although MMP-9 might participate in AV fistula outflow vein dilatation by structural protein digestion, OR was not impaired in the MMP-9^−/−^ mice AV fistula venous segment. Instead, MMP-9 deficiency restricted α-SMA(+) cells from migrating to the neointima area and increased the lumen size of the AVF outflow vein.

Inflammation is a major biological process that influences vascular remodeling of the AV fistula venous segment [7]. In this study, whole transcriptome RNA-seq data analysis of the perioperative AV fistula and contralateral control vein with differential gene expression and functional enrichment revealed that four out of the top 10 upregulated GO terms influenced inflammation, which was confirmed by the elevated expression of multiple proinflammatory cytokines and chemokines. The inflammation in AV fistula could be due to the uremic environment, local surgical trauma, or hypoxia [7]. Inflammation can increase MMP-9 expression. Uremia in CKD conditions increases systemic inflammation. It also increased MMP-9 expression in not only the kidney, but also vascular tissues [41,42,43]. Moreover, uremic toxins induce MMP-9 in vascular smooth muscle cells [44]. We have previously reported that MMP-9 mRNA expression in the AV fistula venous segment was elevated in CKD mice, which was reversed after applying oral charcoal adsorbent to reduce uremic toxin levels [31]. In addition to uremic toxin effects, local trauma and hypoxia due to surgery lead to inflammation in the AV fistula. Inflammatory cells, including neutrophils, macrophages, and lymphocytes, infiltrate the vessel wall [45] and are sources of MMP-9, which in turn facilitates local leukocyte accumulation [46,47]. Proinflammatory cytokine, such as monocyte chemotactic protein-1 (MCP-1), TNF-α, and interleukin 6 (IL-6), levels are also elevated in the AV fistula venous segment [22,48], which is positively associated with MMP-9 expression in vascular smooth muscle cells [49,50,51]. In addition, perivenous adipose MCP-1 and IL-6 levels are inversely related to early postoperative AV fistula veinous segment diameter changes [52]. MCP-1 knockout reduces AV fistula venous thickness and improves fistula patency, while TNF-α induces neointima after arterial injury via the TNF-α/MMP-9 axis [28]. These results support the notion that inflammation may play an important role in neointima formation in the AV fistula probably through MMP-9 action. A relationship between MMP-9 and inflammation has also been suggested in myocardial infarction and MMP-9 knockout may reduce inflammatory cytokines by suppressing inflammatory cell infiltration [53,54]. In this study, GSEA analysis of RNA-seq data using the Hallmark gene set collection revealed negative enrichment of inflammation-related gene sets in AV fistula venous segment of MMP-9^−/−^ mice, which represents suppressed inflammatory response in AV fistula by blocking MMP-9. We also found reduced Mac2(+) macrophage and CD45(+) cell infiltration in the neointima with attenuated TNF-α, IL-6, and MCP-1 expression. Furthermore, IL-6 and MCP-1 expression increased in MMP-9-treated MOVAS cells. Both wound healing and transwell migration assays showed that MMP-9 increased MOVAS cell migration, which was inhibited by the MMP-9 inhibitor. Taken together, these findings indicate that MMP-9 knockout significantly suppressed perioperative vascular inflammation in the AV fistula venous segment, which might reduce neointima formation and increase the lumen area.

Hemodynamic changes in the vessel wall increase local MMP-9 production and influence vascular remodeling, particularly under disturbed or oscillating flow [55]. Computational fluid dynamics simulations have indicated that oscillatory wall shear stress (WSS) zones can be located at the juxta-anastomotic vein area of the end-to-side AV fistula, which coincides with the stenotic area distribution clinically [40]. The venous-end-to-arterial-side AV fistula mice model used in this study replicates the flow pattern of common radial–cephalic AV fistulas in patients. The increased MMP-9 expression in the AV fistula venous outflow tract reflected disturbed flow, and the beneficial effects of MMP-9 knockout in attenuating neointima demonstrate its role in neointima genesis under disturbed flow. On the contrary, IEL dilation in the AV fistula venous segment was not significantly affected by MMP-9 deficiency 4-weeks post creation, despite no compensatory increase in MMP-2 mRNA expression in our results. The response of MMP-2 expression is consistent with the artery injury model in MMP-9 knockout mice by Cho et al. [56], and this may be also due to the higher increase in MMP-2 expression than that in MMP-9 expression (1 week: 20.11 vs. 3.1, 2 week: 143.8 vs. 3.13, 4 week: 8.7 vs. 3.95; Figure 8). In addition, the total MMP-2 protein concentration in normal veins and vein grafts could be approximately 100 times higher than total MMP-9 protein concentration [57]. The role of MMP-2 in vascular remodeling of AVF will best be studied by the use of mice with targeted deletion of this gene. In several animal models, multiple potent elastases, including cathepsin K and S, MMP-2, MMP-9, and MMP-14, have been linked to vascular enlargement under high blood flow after AV fistula creation [58]. Therefore, MMP-9 contribution in the AV fistula venous segment dilation is less than expected and is not the main driving force behind it in this murine AV fistula model.

Janardhanan et al. reported simvastatin reduced AVF venous stenosis through decreasing VEGF-A, MMP-9, and MMP-2 expression on CKD mice [29]. Furthermore, in their recent report, periadventitial local application of simvastatin also had similar effects [59]. In contrary, the administration of doxycycline did not show beneficial effects on venous injury or patency of AVF despite downregulating MMP-9 in the study by Nath et al. [30]. They created AV fistulas on non-CKD mice. In the current study, we also created AV fistulas on CKD mice, and neointima size in AVF was significantly reduced in MMP-9 knockout strain. The discordance among the results of these studies may be owing to the influence of CKD, and different interventions used. CKD has been found to augment MMP-9 expression in AVF [31], and therefore, the inhibition of MMP-9 could have more impact on AVF in CKD mice. Simvastatin influences multiple targets, such as eNOS, endothelin-1, IL-6, IL-1β, and VEGF-A, other than MMP-9 [59,60]. On the other side, doxycycline is a non-selective MMP inhibitor. Both simvastatin and doxycycline have multiple different targets, which potentially confound the interpretation of the effects of MMP-9 on AVF. Since the conflicts of their effects on AVF, we directly targeted MMP-9 and disclosed its beneficial effects in AVF stenosis by reducing local inflammation.

The possible molecular mechanisms underlying the effects of MMP-9 knockout were also analyzed by Western blotting. CD44 and Akt and ERK phosphorylation were suppressed in the AV fistula venous segment of MMP-9^−/−^ mice compared to that in the WT mice. CD44 is a type 1 transmembrane receptor upregulated in vascular endothelial, smooth muscle, and inflammatory cells under inflammatory conditions [61]. In a murine aortocaval fistula model, CD44 expression was elevated after fistula creation. Genetic deletion of CD44 reduced VCAM-1, ICAM-1, and MCP-1 expression, and decreased venous wall thickening. Therefore, CD44 downregulation after MMP-9 knockout partly explains the reduced inflammation in AV fistula. Akt and ERK phosphorylation increases in neointimal lesions of artery injury and AV fistula [62,63,64]. In addition to CD44, Akt and ERK are also downstream of VEGF, angiotensin II, and PDGF [64,65,66,67,68]. Therefore, Akt and ERK pathway suppression may be associated with reduced proliferation, migration, and inflammation of vascular smooth muscle cells [62,64,65].

There are limitations in this study. According to literature review, MMP-9 deficiency may not have significant effects on blood pressure in mice [69]. Since the CKD condition was successfully induced in both the MMP-9 KO and WT mice in our study, we did not evaluate the influence of blood pressure on AVF between MMP-9 knockout and WT mice. However, blood pressure still could potentially affect vascular remodeling.

## 4. Materials and Methods

### 4.1. Animal Study

All animal experimental procedures and protocols were in accordance with the Institutional Animal Care Committee of Taipei Veterans General Hospital (Taipei, Taiwan), and adhered to the Guide for the Care and Use of Laboratory Animals (approval code: IACUC 2017-232, date of approval: 2018-01-10). We used AV fistula model mice with CKD [33,70], which were housed at 22 °C temperature, 41% relative humidity, and 12 h light-dark cycles, with access to food and water ad libitum prior to the experiments. Then, 8-week-old MMP-9^+/+^ (WT) and MMP-9^−/−^ male C57BL/6J mice (Jax lab; Bar Harbor, ME, USA) underwent subtotal nephrectomy to induce CKD as previously reported with some modifications [71]. The upper branch of the left renal artery was ligated with 10-0 nylon (Ethilon; Ethicon; Somerville, NJ, USA) via a flank incision, and the left renal cortex was cauterized to ensure that only 1/3 left kidney was intact. Right-side nephrectomy was performed one week after the first operation.

Four weeks after subtotal nephrectomy, a venous-end to arterial-side AV fistula was created at the right neck as previously described [31], which was modified as described previously [33,70]. Briefly, the mice were anesthetized by intraperitoneally injecting 200 mg/kg 2,2,2-tribromoethanol. After a curved right neck incision, the dorsomedial branch of right EJV and right CCA were dissected. After heparinization with 0.1 IU heparin/g body weight, the dorsomedial EJV branch was anastomosed to right CCA through a 1.0 mm side arteriotomy with 10 to 12 interrupted 11-0 nylon (Ethilon) stitches. After completing the anastomosis, the remaining clamps were removed, and patency was assessed. The skin was closed with a 6-0 nylon running suture.

### 4.2. Tissue Harvesting and Processing

Upon euthanasia, the mice were intraperitoneally injected with 200 mg/kg 2,2,2-tribromoethanol and the AVF was dissected. After thoracotomy, the inferior vena cava was transected, followed by intracardiac perfusion with phosphate-buffered saline (PBS) and then zinc fixative (BD Biosciences; San Jose, CA, USA) for histological examination. The tissue was processed into paraffin, and serial 5-μm sections were made perpendicular to the 1000 µm long AVF venous segment.

### 4.3. Measurement of Serum BUN and Creatinine

The concentrations of serum BUN and creatinine were determined using an autoanalyzer (SpotChem EZ, SP-4430, Arkray; Edina, MN, USA).

### 4.4. Morphometric Analysis

The tissue was stained with Weigert elastin stain (Sigma-Aldrich; St. Louis, MO, USA). Since the most severe neointimal hyperplasia region was within 600 μm downstream of the anastomosis [33], five sections in this region, 100 μm apart and 5 μm thick, were used for analysis. After defining the IEL using Weigert elastin stain, the neointimal lesion and lumen area were measured on digitalized images using ImageJ software.

### 4.5. Collagen Quantification

Collagen content in the neointima of venous vessel was stained with Picro Sirius red (Polysciences; Warrington, PA, USA), analyzed using ImageJ software, and expressed as ratio of positive area after dividing with neointima area of the section [38]. Three sections, 200 μm apart, within the most severe neointimal region were analyzed.

### 4.6. Immunofluorescence and Immunohistochemical Staining

For immunofluorescence analysis, the paraffin tissue sections were cut, mounted, deparaffinized, and rehydrated. After blocking, the sections were incubated with primary antibodies against CD45 and Mac2 (Abcam; Cambridge, UK) in 3% BSA overnight at 4 °C, followed by incubation with the corresponding secondary antibody for 1 h at 25 °C, and nuclei were stained with 4′,6-diamidino-2-phenylindole (DAPI). The CD45- and Mac2-stained cells within the neointima were directly counted in four high-power fields, and the mean numbers of cells were then compared [38]. For immunohistochemical staining, primary antibodies against MMP-9 (Sigma-Aldrich) and α-SMA (Abcam) were used. After overnight incubation, the tissue sections were washed and incubated using EnVision system-horseradish peroxidase-labeled polymer (Dako; Glostrup, Denmark) for 1 h at room temperature. The sections were visualized with 3,3′-diaminobenzidine tetrahydrochloride (Dako) and counterstained with hematoxylin. The MMP-9-stained cells were counted in four high-power fields and compared. The α-SMA-stained area in neointima was analyzed using ImageJ software and expressed as ratio of positive area as Picro Sirius red stain. For both immuno-staining examinations, three sections in the most severe neointimal hyperplasia region, 200 μm apart, were used for each staining.

### 4.7. RNA Extraction and Quantitative PCR Analysis

The AVF venous segment was harvested after perfusion with PBS for RNA extraction. Quantitative real-time PCR was used for the determination of expression of the genes of interest. Total RNA from the vessels was isolated using NucleoZOL (Macherey-Nagel; Duren, Germany) and RNA quality was confirmed by the absorbance 260/280 nm ratio. Reverse transcription was performed using SuperScript III First-Strand Synthesis Supermix (Invitrogen; Carlsbad, CA, USA). SYBR Green Supermix (Bio-Rad Laboratories, Hercules; Berkeley, CA, USA) was used for real-time quantitative PCR, with 35-cycles of amplification using the iQ5 Real-Time PCR Detection System (Bio-Rad Laboratories). Melting curve analysis was used to determine primer efficiency. All samples were normalized using housekeeping gene RNA amplification. The primer sequences are listed in Table S2.

### 4.8. RNA Sequencing, Differential Gene Expression and GSEA

Total RNA for RNA-seq was extracted with NucleoZOL and purified using the Direct-zol RNA Microprep Kit (Zymo Research; Irvine, CA, USA) according to the manufacturer’s instructions. RNA purity and quantification were checked using SimpliNano™-Biochrom Spectrophotometer (Biochrom; Cambridge, UK). RNA degradation and integrity were monitored using a Qsep 100 DNA/RNA Analyzer (BiOptic Inc.; New Taipei, Taiwan). Sequencing libraries were generated using the KAPA mRNA HyperPrep Kit (KAPA Biosystems, Roche; Basel, Switzerland), and index codes were added to attribute sequences to each sample. mRNA sequencing was performed on an Illumina NovaSeq 6000 platform using the standard Illumina RNA-seq protocol. Clear reads were aligned to the mouse genome mm10 assembly using HISTAT2 software (v2.1.0) [72,73]. For gene expression, relative log expression (RLE) normalization and differentially expressed gene (DEG) analysis were performed using DESeq2. GO enrichment analysis of DEGs was conducted using clusterProfiler (v3.10.1). All the genes were ranked by the log2 fold change and the gene set enrichment analysis (GSEA) was performed with 20,000 permutations using the Molecular Signatures Database (MSigDB) Hallmark gene set collection. A gene set was considered to be significantly enriched if its NES had a false discovery rate (FDR) *q*-value < 0.25, and nominal *p*-value < 0.05.

### 4.9. Western Blotting

Western blot analysis was performed as previously described [74]. Briefly, proteins from AV fistula venous segments were extracted using a radioimmunoprecipitation assay (RIPA) buffer containing a protease inhibitor cocktail (cOmplete-Mini, Roche; Indianapolis, IN, USA) after homogenization. Protein concentration was measured using a Bradford assay (Bio-Rad Laboratories; Montreal, QC, Canada). The proteins were separated using sodium dodecyl sulfate-polyacrylamide gel electrophoresis and then transferred to polyvinylidene fluoride membranes. The membranes were probed with primary antibodies against CD44 (Abcam), phospho-Akt, Akt, phospho-ERK, ERK (Cell Signaling Technology; Danvers, MA, USA), and β-actin (Proteintech; Rosemont, IL, USA) at 4 °C overnight. Subsequently, the membranes were incubated with horseradish peroxidase-conjugated secondary antibodies (Jackson ImmunoResearch; West Grove, PA, USA) at room temperature for 1.5 h, and the signals were developed using a West Femto Chemiluminescent Substrate kit (Thermo Fisher; Hudson, NH, USA). Bands were visualized and quantified using a ChemiDoc-It Imaging system (UVP; Cambridge, UK). Data were normalized to β-actin expression.

### 4.10. Cell Culture

The mouse aortic VSMC line (MOVAS) was purchased from the American Type Culture Collection (ATCC, CRL-2797) [75]. The cells were cultured in Dulbecco’s modified Eagle’s medium (DMEM) with 10% fetal bovine serum (FBS; Invitrogen) at 37 °C in a 5% CO_2_ humidified atmosphere. The cells were grown to sub-confluency (60–70%) and starved to synchronize in serum-free DMEM overnight. The cells were then treated with 150 ng/mL MMP-9 (R & D; Minneapolis, MN, USA) in the presence or absence of 50 nM inhibitor (CAS 1177749-58-4; Sigma-Aldrich; St. Louis, MO, USA) for further experiments.

### 4.11. Cell Migration Assay

Migration was determined using wound healing and transwell migration assays [76]. For the wound healing assay, MOVAS was seeded in 24-wells plate and treated with 10 ng/mL MMP-9 (R & D) in the presence or absence of 50 nM CAS 1177749-58-4 (Sigma-Aldrich). A cell-free wound area was created by scratching the cells using a pipette tip. The cell covered area was calculated as the area covered by the migrated cells within the cell-free wound area. For the transwell migration assay, MOVAS cells were seeded onto transwell inserts with 8 μm pore size polyethylene terephthalate membrane (Thermo Fisher Scientific; Waltham, MA, USA) in 24-well plates with MMP-9 in the presence or absence of MMP-9 inhibitor. After 6 h, the medium within the transwell inserts was carefully removed. The cells were fixed with 2% paraformaldehyde, permeabilized with 0.01% Triton X-100 (Sigma-Aldrich), and stained with crystal violet (Sigma-Aldrich). The cells that did not migrate across the transwell membrane were removed by gently wiping with a cotton swab. The migrated cells were viewed and imaged using a phase-contrast microscope. Then, the cells migrated were manually counted in five randomly selected fields and averaged for comparison. 

### 4.12. Statistical Analysis

Statistical analyses were performed using the GraphPad Prism (version 9) software. All data are expressed as the mean ± SEM. The unpaired Student’s *t*-test or one-way analysis of variance (ANOVA) followed by post hoc Bonferroni’s multiple comparison test was used to compare between groups. Unless mentioned otherwise, *p* < 0.05 was considered significant.

## 5. Conclusions

Our study provides evidence supporting the role of MMP-9 in AV fistula neointima formation in CKD condition. Blocking MMP-9 attenuates vascular inflammation and AV fistula venous segment stenosis. These findings warrant further investigation of MMP-9 inhibition therapy for neointima development after AV fistula surgery.

## Data Availability

Not applicable.

## Supplementary Materials

The following are available online at https://www.mdpi.com/article/10.3390/ijms22115448/s1, Table S1: Gene Set Enrichment Analysis with Hallmark Gene Set Collection, Table S2: Primers for Quantitative Real-time Polymerase Chain Reaction.
