# Supplementary material for: MMP-9 Deletion Attenuates Arteriovenous Fistula Neointima through Reduced Perioperative Vascular Inflammation
Source: Int J Mol Sci. 2021 May 21;22(11):5448. doi: 10.3390/ijms22115448 (PMC8196691; doi:10.3390/ijms22115448)
Supplement: Supplementary file 1 [file ijms-22-05448-s001.zip › ijms-1221198-supplementary.pdf]

SUPPLEMENTARY TABLE

Table S1 Gene Set Enrichment Analysis with Hallmark Gene Set Collection

Gene set	Size	ES	NES	p-val	FDR	Category
**WT AVF vs. WT CTL**						
EPITHELIAL_MESENCHYMAL_TRANSITION	190	0.55	2.05	0.00	0.00	Development
G2M_CHECKPOINT	186	0.48	1.80	0.00	0.00	Proliferation
E2F_TARGETS	190	0.43	1.60	0.00	0.01	Proliferation
ANGIOGENESIS	36	0.52	1.56	0.01	0.01	Development
TNFA_SIGNALING_VIA_NFKB	193	0.41	1.53	0.00	0.01	Signaling
INFLAMMATORY_RESPONSE	194	0.40	1.47	0.00	0.03	Immune
ANDROGEN_RESPONSE	96	-0.27	-1.17	0.12	0.24	Signaling
GLYCOLYSIS	193	-0.26	-1.21	0.03	0.19	Metabolic
WNT_BETA_CATENIN_SIGNALING	41	-0.33	-1.22	0.14	0.19	Signaling
ESTROGEN_RESPONSE_LATE	189	-0.28	-1.30	0.01	0.11	Signaling
UV_RESPONSE_UP	148	-0.30	-1.38	0.01	0.07	DNA Damage
ESTROGEN_RESPONSE_EARLY	191	-0.31	-1.44	0.00	0.04	Signaling
REACTIVE_OXIGEN_SPECIES_PATHWAY	44	-0.43	-1.61	0.01	0.01	Pathway
CHOLESTEROL_HOMEOSTASIS	71	-0.43	-1.76	0.00	0.00	Metabolic
HEME_METABOLISM	179	-0.40	-1.89	0.00	0.00	Metabolic
XENOBIOTIC_METABOLISM	187	-0.44	-2.07	0.00	0.00	Metabolic
BILE_ACID_METABOLISM	107	-0.53	-2.34	0.00	0.00	Metabolic
PEROXISOME	97	-0.58	-2.51	0.00	0.00	Cellular Component
FATTY_ACID_METABOLISM	150	-0.72	-3.33	0.00	0.00	Metabolic
OXIDATIVE_PHOSPHORYLATION	190	-0.71	-3.37	0.00	0.00	Metabolic
ADIPOGENESIS	190	-0.75	-3.53	0.00	0.00	Development
**MMP9 AVF vs. WT AVF**						
IL2_STAT5_SIGNALING	193	-0.25	-1.23	0.00	0.18	Signaling
CHOLESTEROL_HOMEOSTASIS	71	-0.29	-1.24	0.04	0.18	Metabolic
GLYCOLYSIS	195	-0.27	-1.24	0.00	0.19	Metabolic
UV_RESPONSE_DN	134	-0.28	-1.28	0.06	0.14	DNA Damage
KRAS_SIGNALING_UP	190	-0.29	-1.36	0.00	0.09	Signaling
HYPOXIA	187	-0.27	-1.41	0.00	0.06	Pathway
APOPTOSIS	155	-0.31	-1.47	0.00	0.04	Pathway
INFLAMMATORY_RESPONSE	194	-0.30	-1.50	0.00	0.04	Immune
TGF_BETA_SIGNALING	52	-0.38	-1.55	0.01	0.02	Signaling
UV_RESPONSE_UP	149	-0.32	-1.55	0.00	0.02	DNA Damage
OXIDATIVE_PHOSPHORYLATION	191	-0.32	-1.58	0.00	0.02	Metabolic
ALLOGRAFT_REJECTION	188	-0.34	-1.69	0.00	0.01	Immune
NOTCH_SIGNALING	31	-0.49	-1.80	0.00	0.00	Signaling
P53_PATHWAY	189	-0.37	-1.81	0.00	0.00	Proliferation
IL6_JAK_STAT3_SIGNALING	85	-0.41	-1.84	0.00	0.00	Immune
MITOTIC_SPINDLE	197	-0.36	-1.85	0.00	0.00	Proliferation
REACTIVE_OXIGEN_SPECIES_PATHWAY	45	-0.48	-1.94	0.00	0.00	Pathway
UNFOLDED_PROTEIN_RESPONSE	103	-0.43	-1.99	0.00	0.00	Pathway
MTORC1_SIGNALING	193	-0.40	-2.00	0.00	0.00	Signaling
G2M_CHECKPOINT	186	-0.42	-2.01	0.00	0.00	Proliferation
DNA_REPAIR	137	-0.48	-2.30	0.00	0.00	DNA Damage

TNFA_SIGNALING_VIA_NFKB	192	-0.47	-2.31	0.00	0.00	Signaling
MYC_TARGETS_V1	190	-0.53	-2.58	0.00	0.00	Proliferation
MYC_TARGETS_V2	56	-0.62	-2.59	0.00	0.00	Proliferation
E2F_TARGETS	191	-0.56	-2.77	0.00	0.00	Proliferation

Gene set enrichment analysis with Hallmark gene set collection for comparison of WT AVF vs. WT CTL and MMP9 AVF vs. WT AVF. Only gene sets with FDR ≤ 0.25 were listed. Size: number of genes in each gene set. ES = enrichment score. NES = normalized enrichment score. p-val = nominal p value. FDR = false discovery rate.

Table S2. Primers for Quantitative Real-time Polymerase Chain Reaction

Gene	Direction	Primer Sequence (5′ - 3′)
TNF-α	Foward	GCCTCTTCTCATTCCTGCTTG
	Reverse	CTGATGAGAGGGAGGCCATT
MMP-2	Foward	AGATCTTCTTCTTCAAGGACCGGTT
	Reverse	GGCTGGTCAGTGGCTTGGGGTA
MMP-9	Foward	CTGGACAGCCAGACACTAAAG
	Reverse	CTCGCGGCAAGTCTTCAGAG
MCP-1	Foward	TGATCCCAATGAGTAGGCTGGAG
	Reverse	ATGTCTGGACCCATTCCTTCTTG
IL-6	Foward	ACGGCCTTCCCTACTTCACA
	Reverse	CATTTCCACGATTTCCCAGA
ICAM-1	Foward	GGCATTGTTCTCTAATGTCTCCG
	Reverse	TGTCGAGCTTTGGGATGGTAG
VCAM-1	Foward	AGTTGGGGATTCGGTTGTTCT
	Reverse	CCCCTCATTCCTTACCACCC
